# Machine learning models including insulin resistance indexes for predicting liver stiffness in United States population: Data from NHANES

**DOI:** 10.3389/fpubh.2022.1008794

**Published:** 2022-09-23

**Authors:** Kexing Han, Kexuan Tan, Jiapei Shen, Yuting Gu, Zilong Wang, Jiayu He, Luyang Kang, Weijie Sun, Long Gao, Yufeng Gao

**Affiliations:** Department of Infection, The First Affiliated Hospital of Anhui Medical University, Hefei, China

**Keywords:** liver cirrhosis, liver stiffness measurement (LSM), insulin resistance, HOMA-IR, METS-IR, machine learning model, NHANES

## Abstract

**Background:**

Prevention and treatment of liver fibrosis at an early stage is of great prognostic importance, whereas changes in liver stiffness are often overlooked in patients before the onset of obvious clinical symptoms. Recognition of liver fibrosis at an early stage is therefore essential.

**Objective:**

An XGBoost machine learning model was constructed to predict participants' liver stiffness measures (LSM) from general characteristic information, blood test metrics and insulin resistance-related indexes, and to compare the fit efficacy of different datasets for LSM.

**Methods:**

All data were obtained from the National Health and Nutrition Examination Survey (NHANES) for the time interval January 2017 to March 2020. Participants' general characteristics, Liver Ultrasound Transient Elastography (LUTE) information, indicators of blood tests and insulin resistance-related indexes were collected, including homeostasis model assessment of insulin resistance (HOMA-IR) and metabolic score for insulin resistance (METS-IR). Three datasets were generated based on the above information, respectively named dataset A (without the insulin resistance-related indexes as predictor variables), dataset B (with METS-IR as a predictor variable) and dataset C (with HOMA-IR as a predictor variable). XGBoost regression was used in the three datasets to construct machine learning models to predict LSM in participants. A random split was used to divide all participants included in the study into training and validation cohorts in a 3:1 ratio, and models were developed in the training cohort and validated with the validation cohort.

**Results:**

A total of 3,564 participants were included in this study, 2,376 in the training cohort and 1,188 in the validation cohort, and all information was not statistically significantly different between the two cohorts (*p* > 0.05). In the training cohort, datasets A and B both had better predictive efficacy than dataset C for participants' LSM, with dataset B having the best fitting efficacy [±1.96 standard error (SD), (-1.49,1.48) kPa], which was similarly validated in the validation cohort [±1.96 SD, (-1.56,1.56) kPa].

**Conclusions:**

XGBoost machine learning models built from general characteristic information and clinically accessible blood test indicators are practicable for predicting LSM in participants, and a dataset that included METS-IR as a predictor variable would improve the accuracy and stability of the models.

## Introduction

Liver cirrhosis is the 14th most common cause of death worldwide, but the fourth most common cause of death in Central Europe (1). Liver cirrhosis has different clinical prognostic stages, with 1-year mortality rates ranging from 1 to 57% (1). Newer research confirmed that although liver stiffness may be reversible in the early stages of liver fibrosis, most patients are asymptomatic until the onset of decompensation (2), which means that the vast majority of patients unconsciously miss the optimal stage of management. Therefore, it is important to obtain timely information on the stiffness of the patient's liver.

Liver biopsy is the gold standard for the diagnosis of liver fibrosis, but the invasive nature of the test has limited its widespread use (3, 4). Therefore, it seems appropriate to re-evaluate the diagnostic performance of other emerging non-invasive tools. In recent years, there has been considerable interest in liver ultrasound transient elastography (LUTE). The principle of LUTE is the stiffness of the tissue being examined in response to an applied mechanical force (compression or shear wave) (5). Although liver ultrasound transient elastography (LUTE) has been widely used as a non-invasive method to detect liver fibrosis. However, due to factors such as affordability, disease awareness and uneven distribution of healthcare resources, LUTE may only be available at higher levels of healthcare facilities. Many people in remote areas may only have access to the most basic public health services and not to LUTE screening (6). Therefore, it is a matter of concern how to identify alterations in liver stiffness in an early stage through a simpler method.

Until then, the non-invasive diagnostic score for liver fibrosis has provided much help to clinicians (6), but many scholars believe that classical scores like AST/platelet ratio index (APRI), Fibrosis-4 (FIB-4) and Fibrotest may only be of their advantage in diagnosing advanced liver fibrosis and still have limitations in differentiating between early and mid-stage liver fibrosis (7, 8). As a result, researchers have been attempting to use new markers or scoring systems for the prediction of liver fibrosis, and the correlation between insulin resistance and liver stiffness has attracted attention as the studies of factors influencing liver fibrosis-related factors have become more sophisticated (9). A growing number of studies have demonstrated a positive correlation between insulin resistance-related indexes and the degree of liver fibrosis (10, 11), However, most previous studies were still limited to demonstrating that insulin resistance may be a risk factor for altered liver stiffness (12–14). Calapod et al. (15) previously used homeostasis model assessment of insulin resistance (HOMA-IR) to develop a Logsitic regression model for predicting the development of severe liver fibrosis in diabetic patients. However, this study, which focused first on participants with NAFLD, not only failed to quantitatively fit liver stiffness but also had unsatisfactory predictive performance as a conventional predictive model. In addition, HOMA-IR index is a classical indirect method of assessing insulin resistance, but it is susceptible to the accuracy of insulin measurements and is poorly reproducible (16). Metabolic score for insulin resistance METS-IR is a recently developed index that aims to be a practical and effective alternative biomarker of insulin resistance (IR). METS-IR is insulin-independent and studies have shown that it is superior to other non-insulin-based indicators of insulin resistance and has the advantage of being stable and reproducible (17).

Unlike traditional machine learning models, XGBoost is an integrated learning algorithm. It uses decision trees as weak learners and in order to perform the gradient descent process it calculates the loss and adds a decision tree to the model to reduce the loss and thus correct the parameters. The number of decision trees is the same as the number of iterations (n-rounds) of the algorithm. Because each decision tree contributes a different value, the final output of XGBoost is given by the mean of the predicted values (weighted) made by all the individual trees (18). In addition, for samples with missing eigenvalues, XGBoost can automatically learn their segmentation direction to achieve the best prediction (19). XGBoost machine learning model is maturing as an artificial intelligence algorithm in the field of medicine, empowering researchers to create models for diagnosis, treatment, management, etc., which can be used to great effect in practice (20–22).

In summary, the aim of this study was to build models capable of predicting liver stiffness using clinically easily accessible information such as data on general characteristics of participants and blood test indicators. In order to improve the fitting efficacy of the prediction model, we opted to build XGBoost machine learning models and compare the fitting efficacy of the original dataset, the dataset containing HOMA-IR, and the dataset containing METS-IR.

## Methods

### Data source

The NHANES program is published by the National Center for Health Statistics (NCHS) and is designed to assess the health and nutritional status of the United States population through information from questionnaires, physical examinations, and laboratory tests. The NHANES program obtains a nationally representative sample of approximately 5,000 individuals per year through a complex, multi-stage sampling design and updates the database every 2 years. NCHS Research Ethics Review committees endorsed the NHANES survey protocol, an informed written consent form was provided to all participants, and all information in the database was available to the public (https://wwwn.cdc.gov/nchs/nhanes/Default.aspx), making our research ethics review exempt.

### Participants

**Figure 1:**
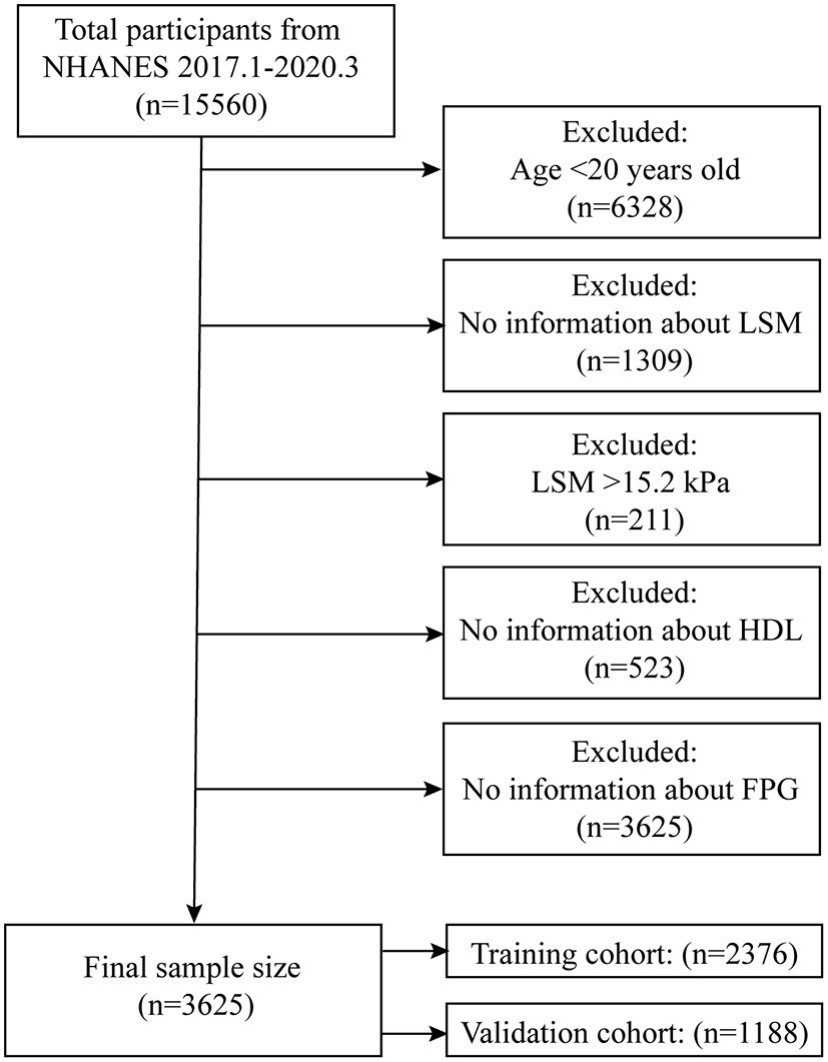
Flow chart for participants.

The NHANES working group has been collecting information on participants' LUTE since 2017 and the NHANES program suspended field operations in March 2020 due to the coronavirus disease 2019 (COVID-19) pandemic, so the time interval for our study was January 2017-March 2020. Within this time range we were able to obtain complete LSM data for participants. A total of 15,560 participants took part in the survey, and we excluded participants younger than 20 years (*n* = 6,328) and those without complete LUTE information (*n* = 1,309). Previous study has graded liver stiffness based on LSM measured by LUTE (F2:8.2kPa; F3:9.7kPa; F4: 13.6kPa) (23). In the data where the LSM exceeded 8.2kPa, while the difference in LSM from F4 to F2 was 3.9kPa (F4-F3) and 1.5kPa (F3-F2), respectively. To more strictly limited the difference between the fitted LSM and the actual LSM, we selected 1.5 kPa as the difference tolerance value. Based on the characteristics of the LSM distribution (Supplementary materials), we finally defined the maximum value of LSM for participants that entered the models as 15.2 kPa, for which we excluded participants with LSM >15.2 kPa (*n* = 211). To obtain complete information on insulin resistance-related indexes, we excluded participants who lacked high-density lipoprotein (HDL) (*n* = 523) and fasting plasma glucose (FPG) (*n* = 3,625). The final total sample size for our study was 3,564, and we subsequently randomly split the total sample population into a training cohort (*n* = 2,376) and a validation cohort (*n* = 1,188) in a 3:1 ratio (Figure 1).

### Liver stiffness measurement

Transient elastography is a widely used and validated technique for the quantitative assessment of tissue stiffness. It is considered a reliable and non-invasive method for assessing liver fibrosis (24, 25). LUTE is able to measure the speed of mechanically generated shear waves through the liver to obtain a measure of liver stiffness, which at a certain level can be a marker for the diagnosis of liver fibrosis (26). LUTE was performed by trained health technicians at the NHANES Mobile Examination Centre (MEC). The participants' LSM was measured using the FibroScan®, which was equipped with medium or extra-large probes to perform the examination. During the examination, 30 measurements were taken for each participant using the medium-sized (M) or large-sized (XL) probes. The medium-sized probe was used first, unless the manufacturer's instructions recommended the use of the large probe. The displacement due to shear waves was tracked and measured using a pulsed echo ultrasound acquisition algorithm. The velocity of the shear wave is directly related to the hardness of the tissue; the harder the tissue, the faster the shear wave propagates. Using Young's modulus, velocity was converted to liver stiffness and expressed in kilopascals. Participants were kept in the supine position throughout the examination. Participants were excluded from participation in the examination if they were unable to lie on the examination table, were pregnant at the time of the examination, had an electronic medical device implanted, were wearing a bandage or had damage to the measurement site. Detailed instructions for this were available on the NHANES website.

### Insulin resistance-related indexes

In this study, HOMA-IR and METS-IR were calculated by the following formulas: HOMA-IR = fasting insulin (uIU/ml) × 360/[fasting glucose (mg/dl)-63] (27); METS-IR = LN [2 × fasting glucose (mg/ml) + fasting triglycerides (mg/dl)] × body mass index (BMI) (kg/m^2^) / LN [high-density lipoprotein (HDL) (mg/dl)] (28). BMI was obtained based on participants' height (m) and weight (kg), calculated as BMI = kg/m^2^. The conditions under which the above indicators were measured by NHANES did not change during the time period of our study.

### Other predictive variables

The predictive variables used to develop the machine learning models in this study consisted mainly of participants' general characteristics information, body examination data and laboratory examination data. The general characteristics information included participants' demographic information [e.g., age, gender, education level, ratio of family income to poverty (PIR), etc.], lifestyle behaviors (e.g., frequency of alcohol consumption, smoking status, sedentary minutes, etc.) and medical conditions (e.g., hypertension, diabetes, hepatitis, etc.). Information on medical conditions was obtained from the hypertension questionnaire (ever been told you have hypertension, age at diagnosis of hypertension, medication used for hypertension treatment), diabetes questionnaire (ever had diabetes, age at diagnosis of diabetes, medication used for diabetes treatment), viral hepatitis questionnaire (ever had hepatitis B, ever been treated for hepatitis B, ever had hepatitis C, ever been treated for hepatitis C), physical activity questionnaire (sedentary activity time). Body examination data included waist circumference (cm), hip circumference (cm), systolic pressure (mmHg), diastolic pressure (mmHg) and BMI. Blood pressure was measured using an oscillometric device and the details of measurement and quality control were available on the NHANES website. Indicators for blood tests (Supplementary Table 1) were collected from laboratory data and all participants were asked to fast for 9 h and assessed by staff for fasting status before blood samples were drawn. NHANES only analyzed samples that met the conditions for laboratory testing. The methods and conditions of NHANES for the measurement of these indicators did not change during the time period of this study.

### Definition of datasets

The LSM served as the final target to be fitted in our machine learning models, and the other variables mentioned above were included as predictor variables in the models. We build three datasets based on the different predictor variables incorporated. The three datasets were named Dataset A (without the insulin resistance-related indexes as predictor variables), Dataset B (with METS-IR as a predictor variable) and Dataset C (with HOMA-IR as a predictor variable). In a dataset that included an index related to insulin resistance, the variables that appeared in the formula would no longer be included separately in the dataset.

### Statistical analysis

Analysis of all data in this study was performed in R (http://www.R-project.org) and EmpowerStats (http://www.empowerstats.com). Continuous variables were expressed as mean ± standard deviation (SD) and statistical variables were expressed as percentages. Missing continuous predictor variables were treated as follows: when the missing value was < 5% of the total sample, the mean was used, otherwise the continuous variables were grouped and the missing values were named “Unclear group.” When the missing values were present in the statistical predictor variables, they were set to “Unclear group.” XGBoost machine learning models were used to predict the participants' LSM. To improve the prediction performance, we used 100 iterations (n-rounds = 100) of the cross-validation process in this study. To prevent overfitting, we eliminated concerns about collinearity between predictor variables based on the principle of regularization and set the following parameters to the model: booster = gbtree, objective = reg:linear, learning rate = 0.3, gamma = 5, max depth = 6, min child weight = 1, lambda = 1, subsample = 1, colsample bytree = 1. The mean-squared error (MSE), mean absolute error (MAE), root mean-squared error (RMSE), coefficient of determination (*R*^2^), and Pearson's correlation coefficient (Pearson's r) were used to assess the accuracy of the models. The relative importance of all predictor variables was calculated by obtaining Gain values and plotting the top 20 predictor variables with the greatest influence on the LSM. The relative importance was calculated as (1/Gain value of top1)^*^Gain value of other predictor variables. A Bland-Altman plot was also generated to show the predicted values and 95% agreement limits, the scatter plot was used to show the degree of correlation between the estimated and actual values.

## Results

### Comparison of training cohort and validation cohort information

The final number of participants included in this study was 3,564, with 2,376 in the training cohort and 1,188 in the validation cohort. The mean LSM value for participants was 5.34 kPa (range 1.6–15.2) and not statistically significantly different between both cohorts (*p* = 0.664), and all predictor variables were not statistically different between the training and validation cohorts (Table 1; Supplementary Table 1).

**Table 1:** Comparison of participant characteristics in the training and validation cohorts.

**Characteristics**	**Training cohort**	**Validation cohort**	***P*-value**
Sample size	2,376	1,188	
Age (years)	50.75 ± 17.11	49.94 ± 17.15	0.182
Gender (%)			0.570
Male	48.40	49.41	
Female	51.60	50.59	
BMI (kg/m^2^)	29.57 ± 7.00	29.84 ± 7.35	0.295
Education level (%)			0.332
Less than high school	20.12	17.76	
High school	23.27	24.83	
More than high school	56.57	57.32	
Unclear	0.04	0.08	
PIR (%)			0.966
< 1.35	23.48	23.99	
1.35–3.45	33.04	33.42	
≥3.45	29.71	29.04	
Unclear	13.76	13.55	
Drinking frequency (%)			0.131
Not at all	19.87	17.93	
≤ 1 times per month	28.62	30.98	
≤ 1 times per week	17.51	20.37	
≥2 times per week	13.85	10.77	
Almost daily	6.69	6.65	
Unclear	13.47	13.30	
Smoker (%)			0.065
Yes	41.54	45.29	
No	58.38	54.71	
Unclear	0.08	0	
Still smoking (%)			0.079
Every day	13.38	15.82	
Some days	4.55	4.04	
Not at all	23.61	25.42	
Unclear	58.46	54.71	
Age started smoking (years) (%)			0.082
< 18	20.12	21.13	
≥18	21.42	24.16	
Unclear	58.46	54.71	
Hypertension (%)			0.245
Yes	36.83	39.65	
No	63.05	60.19	
Unclear	0.13	0.17	
Age of hypertension (years) (%)			0.072
< 40	10.44	13.22	
40–60	18.35	19.02	
≥60	8.04	7.41	
Unclear	63.17	60.35	
Medication for hypertension (%)			0.248
Yes	33.88	36.28	
No	2.86	3.28	
Unclear	63.26	60.44	
Diabetes (%)			0.738
Yes	14.48	15.74	
No	82.37	81.06	
Borderline	3.11	3.11	
Unclear	0.04	0.08	
Age of diabetes (years) (%)			0.332
< 40	2.69	3.45	
40–60	8.21	9.26	
≥60	3.58	3.03	
Unclear	85.52	84.26	
Taking insulin now (%)			0.119
Yes	4.12	3.37	
No	10.35	12.37	
Unclear	85.52	84.26	
Taking diabetic pills now (%)			0.181
Yes	12.21	14.39	
No	15.74	15.07	
Unclear	72.05	70.54	
Ever told you have hepatitis B (%)			0.202
Yes	1.47	1.18	
No	98.23	98.15	
Unclear	0.29	0.67	
Ever treated for hepatitis B (%)			0.910
Yes	0.29	0.25	
No	0.97	0.84	
Unclear	98.74	98.91	
Ever told you have hepatitis C (%)			0.903
Yes	1.64	1.77	
No	97.94	97.73	
Unclear	0.42	0.51	
Ever treated for hepatitis C (%)			0.848
Yes	0.88	1.01	
No	0.63	0.76	
Unclear	98.48	98.23	
Waist Circumference (cm)	99.98 ± 16.25	100.38 ± 16.74	0.640
Hip circumference (cm)	106.89 ± 13.81	107.11 ± 14.27	0.808
Systolic pressure (mmHg)	124.39 ± 18.60	124.24 ± 18.35	0.816
Diastolic pressure (mmHg)	75.06 ± 11.08	75.44 ± 11.20	0.470
Sedentary activity (min)	375.01 ± 713.93	373.22 ± 657.70	0.493
Median liver stiffness (Kpa)	5.34 ± 1.93	5.34 ± 1.97	0.664
METS-IR	44.19 ± 12.73	44.70 ± 13.20	0.264
HOMA-IR	113.52 ± 144.18	103.53 ± 246.13	0.128

Mean ± SD for continuous variables: P-value was calculated by weighted linear regression model. % for Categorical variables: P-value as calculated by weighted chi-square test.

### Prediction performance of training cohort

**Figure 2:**
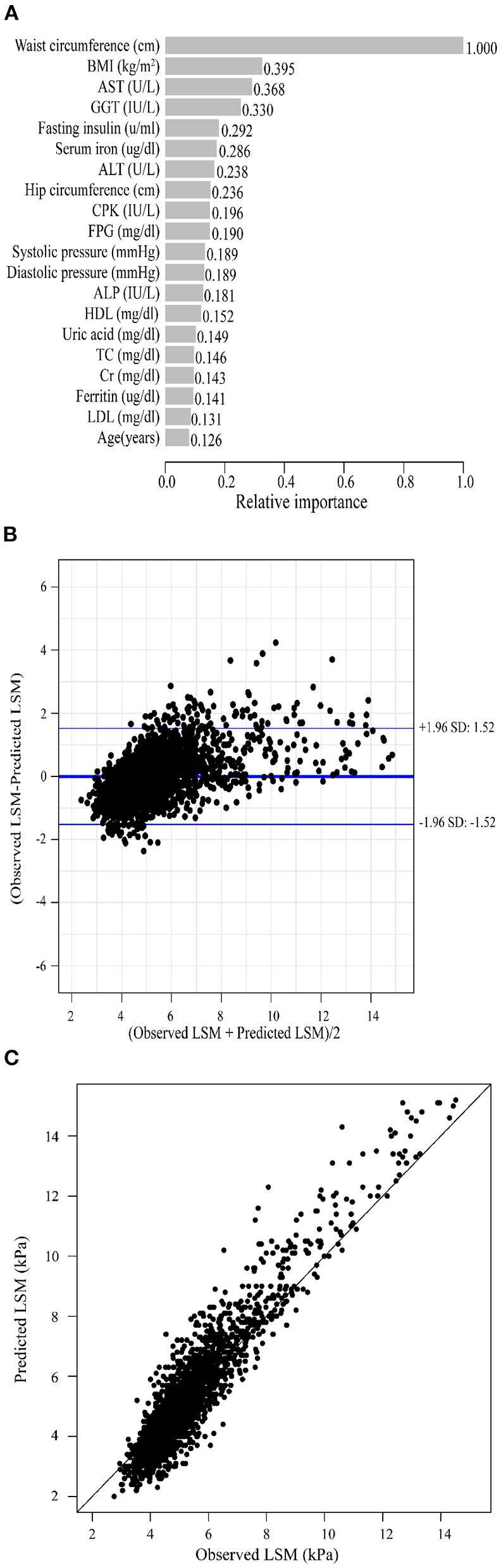
XGBoost machine learning model developed with dataset A in the training cohort. **(A)** Relative importance of the top 20 predictor variables. **(B)** Bland-Altman analysis of estimated LSM (kPa) for real data. The dark blue line in the middle represents the difference between the estimated and true values, and the light blue lines at the top and bottom represent 95% agreement limits of the estimated values. Each black point represents a sample. **(C)** The fitted plot of estimated and true values after XGBoost regression. Each black point represents a sample.

**Figure 3:**
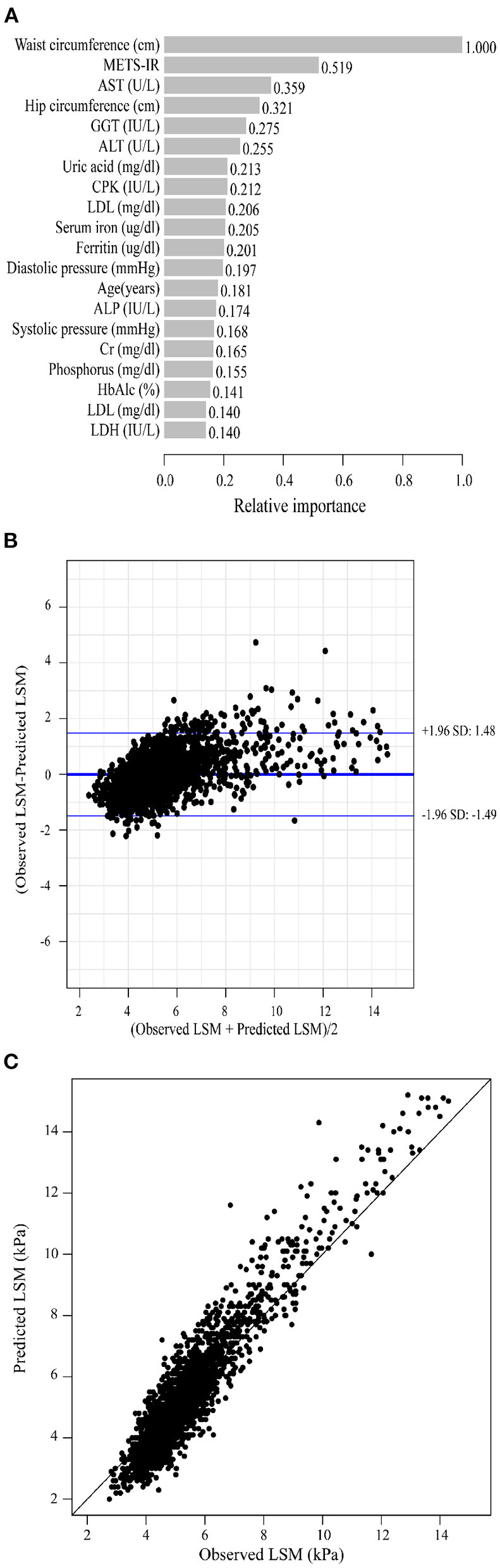
XGBoost machine learning model developed with dataset B in the training cohort. **(A)** Relative importance of the top 20 predictor variables. **(B)** Bland-Altman analysis of estimated LSM (kPa) for real data. The dark blue line in the middle represents the difference between the estimated and true values, and the light blue lines at the top and bottom represent 95% agreement limits of the estimated values. Each black point represents a sample. **(C)** The fitted plot of estimated and true values after XGBoost regression. Each black point represents a sample.

**Figure 4:**
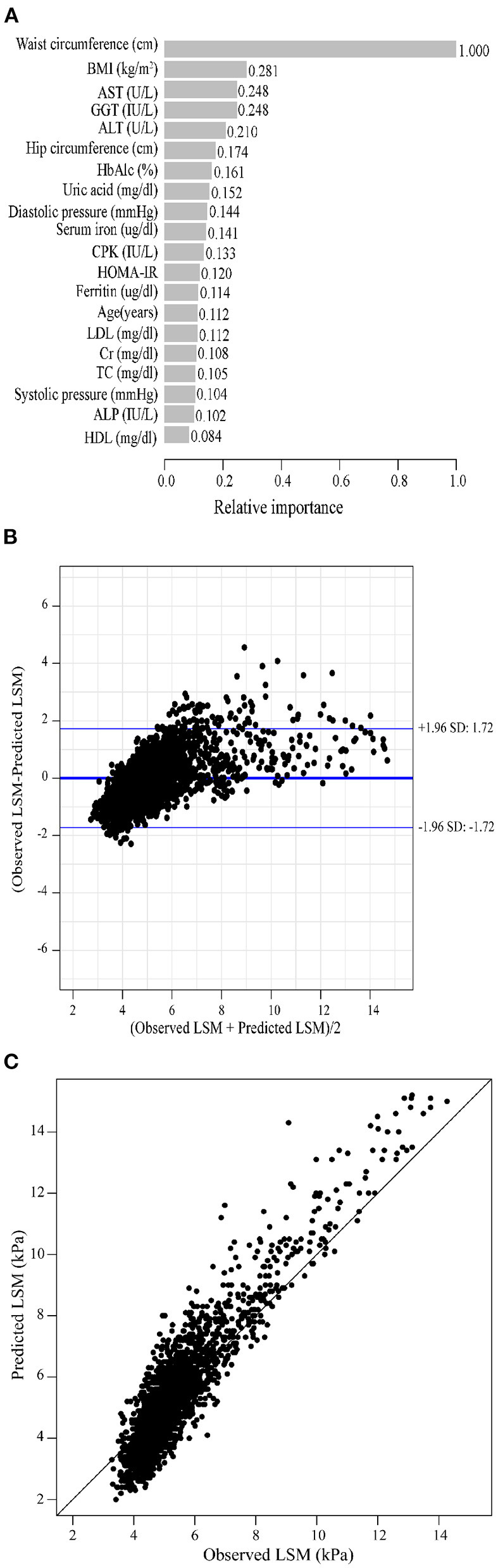
XGBoost machine learning model developed with dataset C in the training cohort. **(A)** Relative importance of the top 20 predictor variables. **(B)** Bland-Altman analysis of estimated LSM (kPa) for real data. The dark blue line in the middle represents the difference between the estimated and true values, and the light blue lines at the top and bottom represent 95% agreement limits of the estimated values. Each black point represents a sample. **(C)** The fitted plot of estimated and true values after XGBoost regression. Each black point represents a sample.

As previously described, we constructed XGBoost machine learning models with three datasets (datasets A, B, and C). We summarized the fitted LSM and the measured LSM values, and the mean values of the LSM produced by the models fitted to the three datasets were almost identical. The minimum values of the LSM produced from the fits using datasets A and B were closer to the actual LSM, and the maximum values of the LSM fitted to dataset B were closest to the actual LSM maximum (Supplementary Table 2). Waist circumference possessed the greatest relative importance in the XGBoost models generated for all three datasets, and the relative importance of METS-IR was more pronounced in the datasets that included insulin resistance-related indexes (Figures 2A, 3A, 4A).

Bland-Altman analysis showed that the difference between the fitted LSM and the measured values was close to 0 for all three models, but the standard deviation of dataset C was larger. The limits of agreement (95%, 1.96 SD) for datasets A, B and C ranged from (-1.52, 1.52) kPa, (-1.49, 1.48) kPa and (-1.72, 1.72) kPa, respectively (Figures 2B, 3B, 4B; Table 2).

**Table 2:** 95% agreement limits of estimated LSM for three datasets in the training cohort.

**Datasets**	**Difference (Predicted-observed)**	**2.5% Limits**	**97.5% Limits**	**SD**
Dataset A	0.00197	−1.52	1.52	0.76
Dataset B	0.00407	−1.49	1.48	0.74
Dataset C	0	−1.72	1.72	0.86

We evaluated the accuracy and stability of the XGBoost machine learning models, and the dataset that included METS-IR showed the best prediction performance and stability among the three machine learning models (Table 3). The fitted plots after XGBoost regression were shown in Figures 2C, 3C, 4C, where the solid black line indicates the perfectly fit reference line, in which we could observe that most of the predicted values in the three machine learning models were scattered around the reference line.

**Table 3:** Evaluation metric values in the training cohort.

**Datasets**	**MSE**	**MAE**	**RMSE**	***R^2^***	**Pearson's r**
Dataset A	0.58	0.58	0.76	0.86	0.93
Dataset B	0.55	0.57	0.74	0.87	0.93
Dataset C	0.74	0.67	0.86	0.83	0.91

### Prediction performance of the validation cohort

Under the same conditions, we validated the XGBoost machine learning models described above using the validation cohort. In the summary table of fitted and actual information, it was seen that the mean value of the fitted LSM for dataset B was closest to the actual LSM (Supplementary Table 3). The relative importance of METS-IR for fitting LSM remained greater than that of HOMA-IR in the datasets containing insulin resistance-related indexes (Supplementary Figures 1A, 2A, 3A). Bland-Altman analysis suggested that the dataset containing METS-IR still had the best fitting performance for LSM, with the limits of agreement (95%, 1.96 SD) of (-1.56, 1.56) kPa, and the dataset A was a better fit for the LSM than dataset C, with the limits of agreement (95%, 1.96 SD) that was (-1.59, 1.59) kPa (Table 4; Supplementary Figures 1B, 2B, 3B). The values of the evaluation metrics were listed in Table 5. Machine learning model constructed from the dataset containing METS-IR outperformed other datasets in terms of accuracy and stability. The machine learning model developed from the dataset containing METS-IR had the best predictive performance for LSM. The fitted plots from the XGBoost regression were shown in Figure 2C, with a larger coefficient between the fitted LSM and the true value for the dataset containing METS-IR (Table 5; Supplementary Figures 1C, 2C, 3C).

**Table 4:** 95% agreement limits of estimated LSM for three datasets in the validation cohort.

**Datasets**	**Difference (Predicted-observed)**	**2.5% Limits**	**97.5% Limits**	**SD**
Dataset A	0.00002	−1.59	1.59	0.80
Dataset B	0	−1.56	1.56	0.78
Dataset C	0	−1.78	1.78	0.89

**Table 5:** Evaluation metric values in the validation cohort.

**Datasets**	**MSE**	**MAE**	**RMSE**	***R^2^***	**Pearson's r**
Dataset A	0.64	0.61	0.79	0.85	0.92
Dataset B	0.61	0.61	0.78	0.87	0.93
Dataset C	0.79	0.69	0.89	0.83	0.91

## Discussion

The normal human liver is soft and elastic, and an increase in liver stiffness only occurs when the liver develops on the basis of chronic substantial injury, a sustained activated inflammatory response and fibrosis formation, with liver fibrosis forming by the end stage (29). This suggests that liver fibrosis is not a single disease, but a common pathological change caused by the development of many chronic liver diseases (30). Globally, the most common causes of cirrhosis are non-alcoholic fatty liver disease (NAFLD) (60%), Hepatitis B virus (HBV) (29%), Hepatitis C virus (HCV) (9%) and alcohol-related liver diseases (ALD) (2%) (31). In European countries, the median prevalence of cirrhosis is 833/100,000, but data on the prevalence of cirrhosis in other regions are scarce, especially in areas with limited healthcare resources (32), which could mean that the global economic and healthcare resource challenge of cirrhosis is grossly underestimated. Although liver biopsy can give a definitive answer to a patient's liver stiffness, many factors limit the acceptance of this test to a wider group of patients, especially those with early liver fibrosis without any clinical symptoms. In order to overcome the limitations of liver biopsy, non-invasive techniques for assessing liver stiffness are now becoming increasingly popular. However, even classical scores such as APRI and FIB-4 still have their limitations, for example, both scores are biased toward the pathogenic microorganism causing cirrhosis (hepatitis C virus), APRI may have insufficient diagnostic value in comparative studies of various scores (33), and FIB-4 has not yet been fully validated in all causes of liver fibrosis (e.g., autoimmune liver disease) (34). In contrast, in our study, participants with or without a previous history of liver conditions were able to enter models.

Liver ultrasound transient elastography is a test that allows assessment of tissue stiffness and permits non-invasive evaluation of liver fibrosis (35), which does not serve every patient with early liver fibrosis due to many factors (e.g., economic factors, geographical factors, patient's perception of the disease, etc.). With the spread of artificial intelligence in medicine, the exploration of the unknown using AI algorithms is becoming increasingly possible, and standing on the shoulders of those who have gone before us, we aimed to use simple information to make predictions about the stiffness of a patient's liver. To our knowledge, there was still no relevant study on quantitative prediction of individual liver stiffness. Atsawarungruangkit et al. (36) had used a machine learning model to predict non-alcoholic fatty liver, but in contrast to their study, our machine learning model did not exclude specific patient-based diseases and could be generalized in a wider population.

Based on previous studies, the LSM cut-off values for cirrhosis severity classes F ≥ F2, F ≥ F3 and F = F4 were 8.2kPa, 9.7kPa and 13.6kPa, respectively (23). In this study, the mean of the differences between the LSM estimates and the actual values produced by our fit using dataset B was 0.047 kPa, and the 95% agreement limits were tightly controlled to within 1.5 kPa. It suggested that the XGBoost machine learning models we developed were not only capable of quantitatively predicting LSM in participants, but also had good discriminative power when grading the severity of liver fibrosis.

It is well known that liver fibrosis can lead to the development of insulin resistance (IR), as liver steatosis may interfere with the function of hepatocytes, particularly their ability to respond to changes in insulin levels leading to the development of IR (25). At the same time, IR can induce the accumulation of hepatic lipids and the production of reactive oxygen species (ROS), and these metabolites can indirectly activate stellate cells and initiate cellular signaling cascades that trigger the development of liver fibrosis (24). The potential mechanism between IR and liver fibrosis could explain the better fitting performance of the datasets containing METS-IR on participants' LSM in this study. In particular, METS-IR has a high relative importance in machine learning models. The most common direct measure of insulin resistance is the high insulin/ normoglycaemic clamp (HEC) technique, which is invasive, complex and impractical. Calapod et al. (15) previously developed a Logsitic regression model using HOMA-IR to predict the development of severe liver fibrosis in diabetic patients. However, HOMA-IR is susceptible to the accuracy of insulin measurements and is poorly reproducible (37). As previously mentioned, METS-IR is insulin independent and diagnostically superior to other non-insulin indexes of insulin resistance (17, 38). In this study, we demonstrated that the dataset containing METS-IR had better fit efficacy for LSM and that the simpler, more accurate and more practical nature of METS-IR makes machine learning models built with METS-IR as predictor variables better applicable.

In our study, we demonstrated the appropriateness of using XGBoost machine learning models for predicting LSM in populations, and also confirmed the advantages of METS-IR for improving the accuracy and stability of the models. However, there were still some limitations to our study. First, some of the information in the medical conditions questionnaire might be subject to recall bias, such as age at diagnosis of hypertension and diabetes. However, all of this information received very low relative importance as predictor variables in the model, so we believe that such a bias is acceptable. Secondly, we would not discount the importance of indices such as APRI, FIB-4, HA, etc., and it would be of great help to our study to have access to this information, unfortunately the NHANES database does not currently contain these data. However, the XGBoost machine learning model uses a monitored learning algorithm and the inclusion of more valuable predictor variables into the model will bring the fitted values closer to the true values (39, 40), suggesting the value of subsequent research to improve and validate the model developed in this study using a cohort containing the above information. Finally, in this study we only validated the machine learning models internally; in order to make them more applicable, we believe that external validation is needed, and this will be the direction of our subsequent research.

## Conclusions

In this study, we demonstrated the feasibility of the XGBoost machine learning model for predicting LSM, and the inclusion of METS-IR as a predictor variable greatly helped to improve the accuracy and stability of the model. The XGBoost machine learning model is similar to a clinician's black box, and the subsequent inclusion of more valuable predictor variables will make the model more worthy of replication.

## Data availability statement

The original contributions presented in the study are included in the article/Supplementary materials, further inquiries can be directed to the corresponding authors.

## Author contributions

Study design: KH. Data analysis and manuscript writing: KT and JS. Manuscript editing: YGu, ZW, JH, LK, and WS. Validation and review: LG. Quality control: YGa. All authors agreed on the journal to which the article was to be submitted, agreed to take responsibility for all aspects of the work, contributed to the article, and approved the submitted version.

## Conflict of interest

The authors declare that the research was conducted in the absence of any commercial or financial relationships that could be construed as a potential conflict of interest.

## Publisher's note

All claims expressed in this article are solely those of the authors and do not necessarily represent those of their affiliated organizations, or those of the publisher, the editors and the reviewers. Any product that may be evaluated in this article, or claim that may be made by its manufacturer, is not guaranteed or endorsed by the publisher.
