# Supplementary figures and images for: Machine learning models including insulin resistance indexes for predicting liver stiffness in United States population: Data from NHANES
Source: Front Public Health. 2022 Sep 23;10:1008794. doi: 10.3389/fpubh.2022.1008794 (PMC9537573; doi:10.3389/fpubh.2022.1008794)

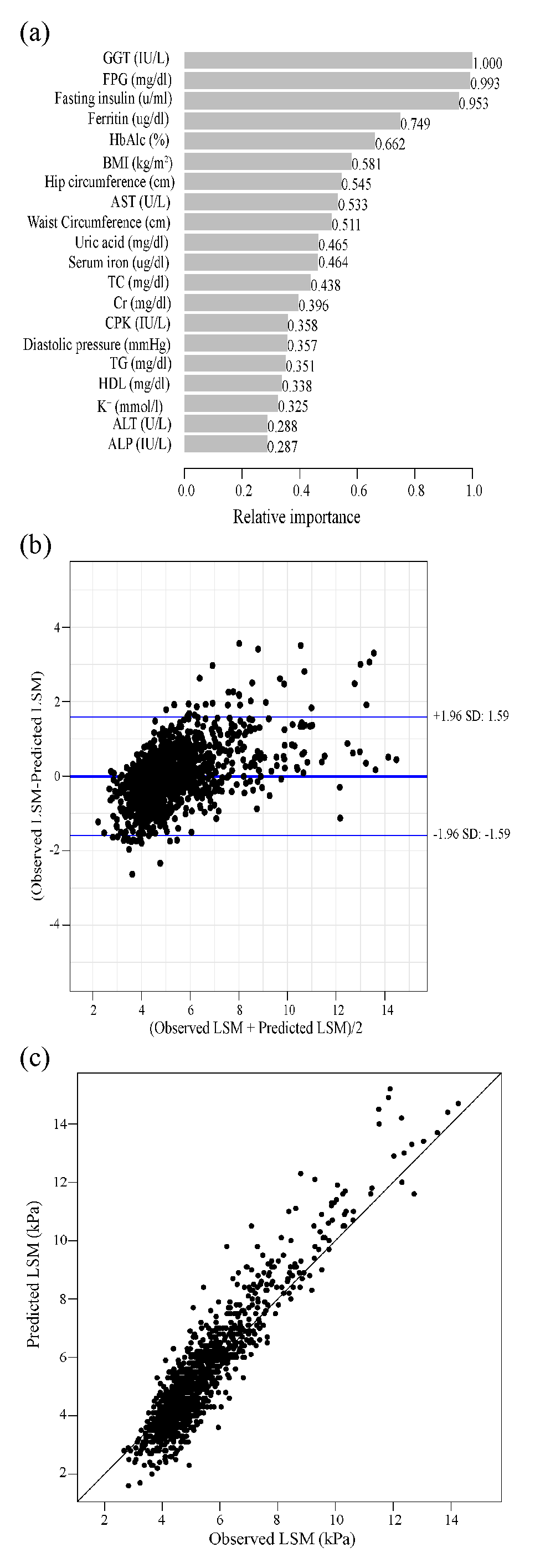

Supplement: Supplementary file 1 [file Image_1.TIF]

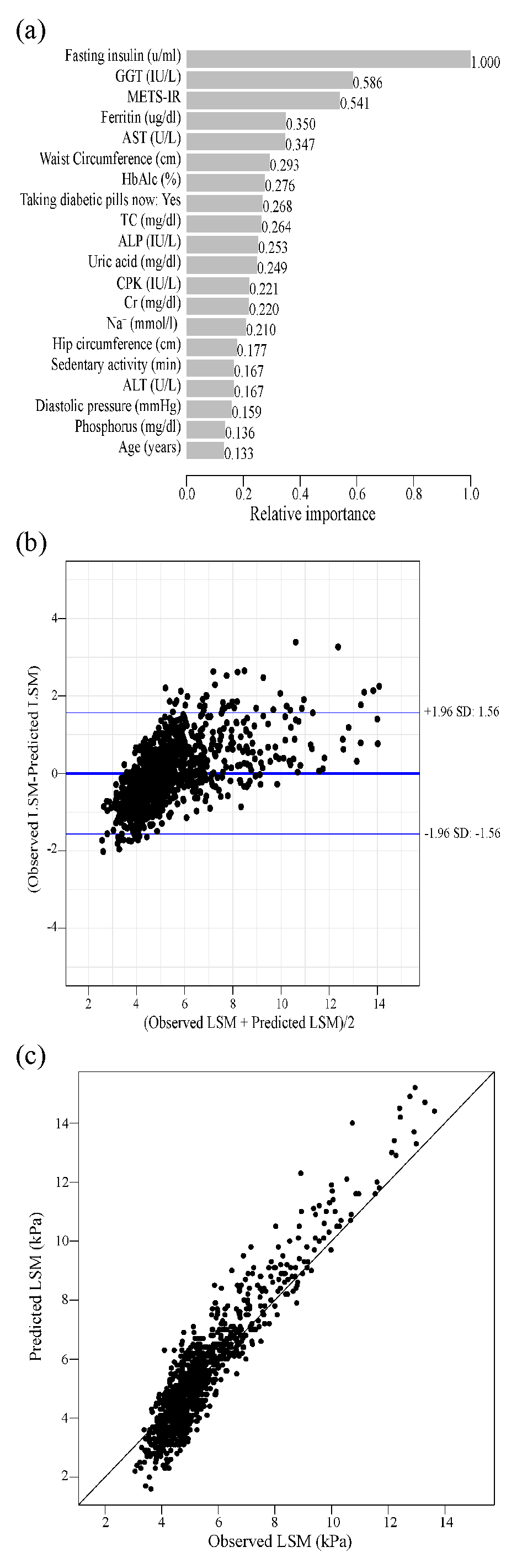

Supplement: Supplementary file 2 [file Image_2.TIF]

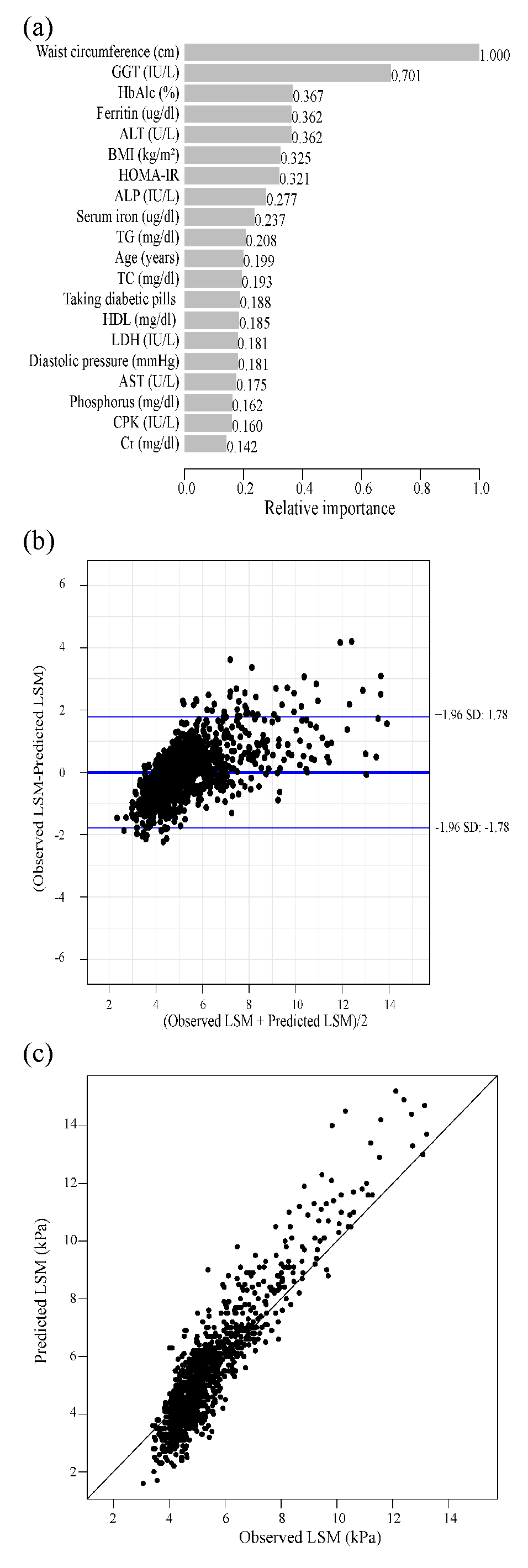

Supplement: Supplementary file 3 [file Image_3.TIF]
