# Supplementary material for: Machine learning models including insulin resistance indexes for predicting liver stiffness in United States population: Data from NHANES
Source: Front Public Health. 2022 Sep 23;10:1008794. doi: 10.3389/fpubh.2022.1008794 (PMC9537573; doi:10.3389/fpubh.2022.1008794)
Supplement: Supplementary file 4 [file Table_1.doc]

**Supplementary table 1.** Comparison of participant blood parameters of in the training and validation cohorts

| Characteristics | Training cohort | Validation cohort | P-value |
| --- | --- | --- | --- |
| Sample size | 2376 | 1188 |  |
| ALT (U/L) | 21.39 ± 15.24 | 23.26 ± 25.48 | 0.120 |
| AST (U/L) | 21.11 ± 10.80 | 22.15 ± 18.35 | 0.295 |
| ALP (IU/L) | 77.34 ± 24.39 | 77.23 ± 24.88 | 0.451 |
| GGT (IU/L) | 30.31 ± 37.53 | 30.91 ± 36.75 | 0.647 |
| TBIL (mg/dl) | 0.49 ± 0.29 | 0.48 ± 0.26 | 0.241 |
| CPK (IU/L) | 165.80 ± 342.03 | 161.25 ± 212.03 | 0.468 |
| ALB (g/dl) | 4.02 ± 0.32 | 4.03 ± 0.33 | 0.499 |
| GLB (g/dl) | 5.85 ± 1.11 | 5.88 ± 1.17 | 0.796 |
| TP (g/dl) | 7.14 ± 0.43 | 7.13 ± 0.44 | 0.260 |
| HDL (mg/dl) | 53.75 ± 15.28 | 53.51 ± 16.78 | 0.213 |
| LDL (mg/dl) | 111.29 ± 35.81 | 110.86 ± 35.80 | 0.710 |
| TC (mg/dl) | 185.14 ± 40.22 | 184.33 ± 40.88 | 0.620 |
| TG (mg/dl) | 125.35 ± 95.43 | 122.82 ± 80.35 | 0.495 |
| Uric acid (mg/dl) | 5.46 ± 1.45 | 5.42 ± 1.42 | 0.418 |
| BUN (mg/dl) | 14.68 ± 5.46 | 14.66 ± 5.64 | 0.790 |
| Cr (mg/dl) | 0.88 ± 0.42 | 0.90 ± 0.51 | 0.544 |
| LDH (IU/L) | 156.73 ± 31.32 | 157.64 ± 34.96 | 0.432 |
| HCO3- | 25.52 ± 2.36 | 25.61 ± 2.32 | 0.255 |
| Phosphorus (mg/dl) | 3.48 ± 0.50 | 3.49 ± 0.54 | 0.563 |
| K+ (mmol/l) | 4.12 ± 0.36 | 4.11 ± 0.35 | 0.530 |
| Na+ (mmol/l) | 140.76 ± 2.46 | 140.76 ± 2.59 | 0.873 |
| Cl- (mmol/l) | 101.74 ± 2.69 | 101.63 ± 2.86 | 0.349 |
| Total calcium (mg/dl) | 9.23 ± 0.35 | 9.23 ± 0.36 | 0.753 |
| Serum iron (ug/dl) | 89.53 ± 37.66 | 90.71 ± 36.07 | 0.298 |
| Ferritin (ug/dl) | 155.27 ± 166.08 | 163.34 ± 175.57 | 0.137 |
| HbAlc (%) | 5.85 ± 1.11 | 5.88 ± 1.17 | 0.549 |
| FPG (mg/dl) | 112.91 ± 37.24 | 113.18 ± 36.95 | 0.837 |
| Fasting insulin (u/ml) | 14.58 ± 22.72 | 13.60 ± 15.00 | 0.178 |

Mean ± SD for continuous variables: P value was calculated by weighted linear regression model.

% for Categorical variables: P value as calculated by weighted chi-square test.

**Supplementary table 2.** Summary of basic information on LSM (kPa) estimated and measured values for the training cohort.

| Datasets | Sample size | Minimum LSM (kPa) | Median LSM (kPa) | Maximum LSM (kPa) |
| --- | --- | --- | --- | --- |
| Dataset A |  |  |  |  |
| Observed | 2376 | 2 | 5 | 15.20 |
| Predicted | 2376 | 2.75 | 5.01 | 14.52 |
| Dataset B |  |  |  |  |
| Observed | 2376 | 2 | 5 | 15.2 |
| Predicted | 2376 | 2.75 | 5.02 | 14.28 |
| Dataset C |  |  |  |  |
| Observed | 2376 | 2 | 5 | 15.20 |
| Predicted | 2376 | 3.11 | 5.01 | 14.38 |

**Supplementary table 3.** Summary of basic information on LSM (kPa) estimated and measured values for the validation cohort

| Datasets | Sample size | Minimum LSM (kPa) | Median LSM (kPa) | Maximum LSM (kPa) |
| --- | --- | --- | --- | --- |
| Dataset A |  |  |  |  |
| Observed | 1188 | 1.60 | 4.90 | 15.20 |
| Predicted | 1188 | 2.67 | 4.94 | 14.26 |
| Dataset B |  |  |  |  |
| Observed | 1188 | 1.60 | 4.90 | 15.20 |
| Predicted | 1188 | 3.06 | 4.90 | 13.63 |
| Dataset C |  |  |  |  |
| Observed | 1188 | 1.60 | 4.90 | 15.20 |
| Predicted | 1188 | 3.07 | 4.97 | 13.21 |

**Supplementary Figure 1.** XGBoost machine learning model developed with dataset A in the training cohort. (a) Relative importance of the top 20 predictor variables. (b)Bland-Altman analysis of estimated LSM (kPa) for real data. The dark blue line in the middle represents the difference between the estimated and true values, and the light blue lines at the top and bottom represent 95% agreement limits of the estimated values. Each black point represents a sample. (c) The fitted plot of estimated and true values after XGBoost regression. Each black point represents a sample.

**Supplementary Figure 2.** XGBoost machine learning model developed with dataset B in the training cohort. (a) Relative importance of the top 20 predictor variables. (b)Bland-Altman analysis of estimated LSM (kPa) for real data. The dark blue line in the middle represents the difference between the estimated and true values, and the light blue lines at the top and bottom represent 95% agreement limits of the estimated values. Each black point represents a sample. (c) The fitted plot of estimated and true values after XGBoost regression. Each black point represents a sample.

**Supplementary Figure 3.** XGBoost machine learning model developed with dataset B in the training cohort. (a) Relative importance of the top 20 predictor variables. (b)Bland-Altman analysis of estimated LSM (kPa) for real data. The dark blue line in the middle represents the difference between the estimated and true values, and the light blue lines at the top and bottom represent 95% agreement limits of the estimated values. Each black point represents a sample. (c) The fitted plot of estimated and true values after XGBoost regression. Each black point represents a sample.
